# Distinct healthy and atopic canine gut microbiota is influenced by diet and antibiotics

**DOI:** 10.1098/rsos.221104

**Published:** 2023-04-26

**Authors:** Hanna Sinkko, Jenni Lehtimäki, Hannes Lohi, Lasse Ruokolainen, Anna Hielm-Björkman

**Affiliations:** ^1^ Department of Equine and Small Animal Medicine, University of Helsinki, Helsinki, Finland; ^2^ Department of Bacteriology and Immunology, Human Microbiome Research (HUMI), University of Helsinki, Helsinki, Finland; ^3^ Department of Medical and Clinical Genetics and Department of Veterinary Biosciences, University of Helsinki, Helsinki, Finland; ^4^ Faculty of Biological and Environmental Sciences, University of Helsinki, Helsinki, Finland; ^5^ Environmental Policy Centre, Finnish Environment Institute, 00790 Helsinki, Finland; ^6^ Folkhälsan Research Center, Helsinki, Finland

**Keywords:** gut microbiome, Western lifestyle, diet, allergic diseases, canine model, pet dogs

## Abstract

The rising trend in non-communicable chronic inflammatory diseases coincides with changes in Western lifestyle. While changes in the human microbiota may play a central role in the development of chronic diseases, estimating the contribution of associated lifestyle factors remains challenging. We studied the influence of lifestyle—diet, antibiotic use, and residential environment with housing and family—on the gut microbiota of healthy and owner-reported atopic pet dogs, searching for associations between the lifestyle factors, atopy and microbiota. The results showed that atopic and healthy dogs had contrasting gut microbial composition. The gut microbiota also differed between two breeds, Labrador Retriever and Finnish Lapphund, selected for our study. Among all lifestyle factors studied, diet was most significantly associated with gut microbiota but only weakly with atopic symptoms. Thus, diet- and atopy-associated changes in the microbiota were not interrelated. Instead, the severity of symptoms was positively associated with the usage of antibiotics, which in turn was associated with the microbiota composition. Urban lifestyle was significantly associated with the increased prevalence of allergies but not with the gut microbiota. Our results from pet dogs supported previous evidence from humans, demonstrating that antibiotics, gut microbiota and atopic manifestation are interrelated. This congruence suggests that canine atopy might be a promising model for understanding the aetiology of human allergy.

## 1. Background

There is substantial evidence suggesting that the rapidly increasing incidence of inflammatory disorders results from the lack of sufficient immune education by diverse microbiota of environmental origin [1,2]. However, as indicated by both indirect evidence and recently discovered molecular mechanisms between dietary patterns and prevalence of allergies, diet can also play an important role [3,4]. The ‘diet hypothesis' emphasizes the association between dietary factors, the gut microbiota and the immune system in the development of allergy [4]. In support of the diet hypothesis, ‘Westernization’ of the human diet, and lifestyle in general, has been associated with dysbiosis in the gut microbiota [5–7]. This disturbed state of gut microbiota can predispose to the development of non-communicable inflammatory disorders such as allergies and asthma [3,8,9].

In addition to dietary components, the gut microbiota can also be influenced by other lifestyle-related factors [10–12]—e.g. via exposure to environmental microbes. However, studies combining both non-dietary effects, such as exposure to rural environment, outdoor living or animal contacts and dietary habits, are limited [13,14]. Furthermore, causation between lifestyle, gut dysbiosis, and inflammatory conditions are hard to determine in humans, due to numerous confounding factors acting to increase inter-individual compositional variation [15]. While human lifestyle is not straightforward to model in laboratory mice, pet dogs share the lifestyle of their owners but have a simpler and shorter lifespan. The diet of companion dogs is generally similar from day to day and thus easy to control, producing less time-dependent and inter-individual variation in the gut microbiota, especially if the diet consists of kibble, the most usual type of diet for dogs. Importantly, the associations between predisposing lifestyle and environmental factors to allergy seem to be analogous between humans and dogs [16].

Patterns in dog feeding reflect the trends in human nutrition [17–19]. Coinciding with ’Westernization’ of human diet (e.g. processed and high-fat, -sugar, -salt) [20], the popular dog-feeding method in Western countries nowadays is based on processed food (kibbles) e.g. [19,21], rich in carbohydrates [22]. This contrasts with the evolutionary adaption of the dog to mostly animal protein and fat [23], and that dogs do not have any nutritional requirement for carbohydrates and what dogs themselves prefer [22]. Concurrently, allergy prevalence among dogs seems to be increasing [24]. The differences in the gut microbiota between atopic and healthy dogs are not widely studied [25,26], in particular considering the concurrent effect of diet. Similarly to humans [27], diet plays a dominant role in affecting gut microbial compositions also in pet dogs [28]. Also, the responses in the canine gut microbiota to inflammatory conditions resemble those of human's [29]. These points of resemblance suggest that diet is likely to be involved also in canine allergy, often referred to as canine atopic dermatitis (CAD), or shortly as canine atopy [30].

Canine AD occurs in 3–15% of dogs, similar to humans [31]. Main symptoms in both species are chronic or chronically relapsing symmetrical skin lesions corresponding to eczematous dermatitis with pruritus. In humans, the areas mainly affected are the scalp, face, neck and flexural surfaces of the extremities. Likewise in dogs, the areas most affected are face (ears, around eyes and mouth), the flexural surfaces of the extremities and spreading from there to the axillae and ventrum [32]. However, to some extent, these diseases also manifest differently in dogs and humans, probably due to differences in immunological responses. For example, allergic dogs mostly have skin- and gut-related symptoms while airway symptoms are rare, as opposed to humans where allergic rhinitis and asthma occur alongside skin- and gut-related symptoms.

Considering the similarities between canine and human AD and the other above-mentioned aspects, dogs appear suitable to study the effects of diet and other lifestyle determinants on gut microbiota and their potential allergy associations, as previously demonstrated [16,33]. Thus, we aimed to investigate the influence of diet, lifestyle and living environment on the gut microbiota of dogs, and whether these are associated with the owner-reported atopy symptoms in dogs. The composition of the dog diet was characterized using a questionnaire: dogs were primarily fed either a heat-processed food rich in carbohydrates (i.e. kibble or dry food), a non-heat-processed food rich in animal proteins and fat (i.e. raw food), or a mix of both. Breed-related variation in microbial composition was estimated by selecting two common breeds, Finnish Lapphunds and Labrador Retrievers, for the study. Our relatively large sample enabled us to reach individuals from different geographical areas, with different lifestyle patterns and either healthy or with atopy symptoms. This in turn facilitated the characterization of lifestyle- and atopy-associated compositional changes in the canine gut microbiota.

## 2. Material and methods

### 2.1. Selection of participants, collection of samples and background information

The study included 155 privately owned pet dogs, of which 34 were mother dogs and the rest their adult offspring. Two dog breeds, Labrador Retriever (*n* = 59) and Finnish Lapphund (*n* = 96), were selected as described in Lehtimäki *et al*. [33]. Shortly, the selected breeds are common in Finland and occur equally in rural and urban areas. Labrador Retrievers are more prone to develop atopy than Finnish Lapphunds [34], facilitating the examination of breed- and atopy-associated changes in microbiota.

Faecal samples were collected by the dog owners at home during a limited period in November 2014 to avoid any seasonal effects. Following detailed instructions, owners sampled a spoonful using a tube with spatula (Sarstedt #80.623.022) from the central part of the faeces, a part that had not touched the ground and was not hard. Samples were placed in the household freezer (−18°C) immediately or within an hour after collection and were later transported as frozen to the laboratory freezer (−20°C). Owners also filled a comprehensive questionnaire, described and validated in Lehtimäki *et al*. [33], where information about atopic symptoms and environmental, lifestyle as well as dietary factors of their dogs were collected.

### 2.2. Sequencing of the bacterial 16S rRNA gene and subsequent bioinformatics

DNA was extracted from faecal samples with QIAamp DNA Stool Mini Kit (Qiagen GmbH, Hilden, Germany) and, as previously described [35], was used as a template in PCR to amplify the V1–V3 regions of the bacterial 16S rRNA gene that was subsequently sequenced as in Lehtimäki *et al*. [33].

The processing of the 16S rRNA gene sequences with the subsequent clustering and annotation of operational taxonomic units (OTUs) was performed as recently described in detail [33]. Briefly summarized, after removing the primers from the raw reads by cutadapt v. 1.9.1. [36], pair-end reads were merged using USEARCH v. 9.2.64 [37] and merged reads quality-trimmed. Sample-specific trimmed reads were dereplicated in VSEARCH v. 2.64 [38]. These sequences were sorted by size, and rare sequences (abundance less than 10) were removed. Chimeras and other artefacts were removed using the unoise2 command [39] and subsequently the reads were mapped to OTUs with 99% similarity threshold using VSEARCH command usearch_global. The representative sequences of OTUs were classified with SILVA rRNA gene sequence database (v. 123) [40,41] using Mothur [42] command classify.seqs with 80% similarity cut-off.

The cumulative-sum scaling [43,44] was used to normalize the processed 16S rRNA gene sequence counts. Potential contaminants were removed after the normalization before further analysis, as described in Lehtimäki *et al*. [33], with the exception that we do not have controls for the sampling performed by dog owners.

### 2.3. Statistical analyses

#### 2.3.1. Variables representing residential environment, atopy, lifestyle, dietary factors and antibiotic use

The large questionnaire information was first simplified as reported in Lehtimäki *et al*. [33]. To summarize it here, we formed continuous variables from the questionnaire data using dimensionality reducing unconstrained methods. The continuous variables were then used in the statistical analyses to determine their effect on the gut microbiota. Each type of multivariate dataset, i.e. land-use types of residential environment, lifestyle factors and owner-reported atopic symptoms, were reduced to one or two summarizing variables using either principal component analysis (PCA; environment) or principal coordinate analysis (PCoA; lifestyle and allergy, based on Gover distance) [33]. The variables are further described in electronic supplementary material, additional file 1.

The formed continuous variables for environment, lifestyle and atopy were further transformed into categorical variables, as previously described [33]. Dogs were classified as living in either a rural or urban environment, having either a rural or urban lifestyle, and being either healthy or atopic, i.e. having owner-reported CAD.

Due to the complexity of canine atopic dermatitis (canine atopy) [45] and the uncertainty of using immunoglobulin E (IgE) as a clinical outcome in dogs [46], several cutaneous symptoms were questioned from dog owners as previously reported [33]. This validated questionnaire [47] was developed at the University of Helsinki by specialists in canine clinical nutrition, disease and dermatology. The answers of atopic symptoms were then simplified with PCoA to represent owner-reported atopic symptoms in general or to rule out them.

Unlike in Lehtimäki *et al*. [33], here the information on dietary factors (appendix S1, questions numbered from 7.1 to 7.4. in Lehtimäki *et al*. [33]) and oral exposure to dirt, i.e. if dogs were allowed to eat or taste plants, soil and faeces, drink muddy water etc. outdoors (appendix S1, question number 8.6 in Lehtimäki *et al*. [33]) were not included in the PCoA of lifestyle factors. The aspects related to oral exposure to dirt were summarized in a separate PCoA based on Gower distance (electronic supplementary material, additional file 1). Related to diet, the dog owners reported in detail how they fed their dogs, during a period of seven days. To control the accuracy of the diet diaries, the owners were also asked to select one of nine alternatives (reported in appendix S1, question number 7.1 in Lehtimäki *et al*. [33]) to describe their dog's diet. If these answers did not correspond well with the other given information or if there were other unclarities, these owners (*n* = 19) were approached with further questions by email.

Based on the given information, we divided the used diets into three categories: (i) A non-heat-processed (NHeP) low carbohydrate (LC) diet with raw (R) products (NHeP-LC-R), (ii) a heat-processed (HeP) high carbohydrate (HC) dry (D) kibble diet (HeP-HC-D), and (iii) a heat-processed (HeP) high carbohydrate (HC) moist (M) food diet (HeP-HC-M). The third category, i.e. HeP-HC-M diet, included canned or home-cooked food, or different heat-processed ’sausage packed’ products, produced for dogs. For each dog, we estimated the percentage of food items belonging to HeP-HC-D, NHeP-LC-R and HeP-HC-M diets by their volume (ml) or weight (g), depending on which measure the dog owner had used in their dogs' food-diaries. As the most significant dietary factor, NHeP-LC-R was selected to be used in further statistics (electronic supplementary material, additional file 1, figure S2). Because actual quantities of different food types were only roughly reported by owners, we also approximated the percentage of NHeP-LC-R food into categories that described whether NHeP-LC-R items were used in the diet mostly/solely, partly or never. Additionally, we determined whether the diet included nutraceuticals such as probiotic and fat (predominantly omega-3 oil) products daily, sometimes or never.

The use of antibiotics was asked in our original questionnaire [33], reporting if skin symptoms of dogs has been treated with antibiotics and if symptoms eased. Eighty-six dog owners passed this question, but also other questions concerning different medication used for skin symptoms. They most likely passed the questions because they reported no (*n* = 53) or very mild skin symptoms (*n* = 33) such as little skin itchiness (*n* = 14) or skin infections (*n* = 2) and never redness or eczema (*n* = 67). Indeed, our analysis categorized only one of these dogs as atopic, as previously described [33]. Thus, we assumed that these 86 dogs had not used antibiotics. In the statistical analyses, we used a categorical variable whether dogs have been treated with antibiotics (once or more) or not.

#### 2.3.2. Relationships between the gut microbiota, atopic symptoms, environmental and lifestyle factors

In examining the variation in gut microbial composition, i.e. betadiversity (defined by Bray–Curtis dissimilarity), distance-based redundancy analysis (db-RDA) in the vegan package [48] was used to assess the role of different explanatory factors (electronic supplementary material, additional file 1, figure S1). In the first db-RDA, illustrating overall patterns, dog health (dogs defined as atopic or healthy) and breed were used as constraining (categorical) variables. In the second db-RDA, demonstrating the effect of diet on the gut microbiota, the percentage of NHeP-LC-R items in dogs' daily intake was used as a constraining variable, after adjusting for the effects of nutraceutical use (probiotics and fat products) and HeP-HC-M diet. The remaining residual variation was then constrained by the percentage of NHeP-LC-R products. HeP-HC-D was not considered as it correlated strongly with NHeP-LC-R (Pearson correlation −0.80, *p* < 2.2 × 10^−16^). In the third db-RDA, the severity of atopic symptoms was used as the explanatory variable. Overlapping effects of NHeP-LC-R and especially antibiotics were visualized along with the ordination. Finally, we tested whether urban and rural land-use and lifestyle patterns and oral exposure to dirt affected the microbial dissimilarity. The significance and effects of the studied variable was estimated by multiple regression on distance matrices (MRM), using 9999 permutations in the vegan package [48].

To support the compositional changes observed in db-RDA analyses, we specified those OTUs that significantly varied between the different groups, e.g. atopic and healthy dogs, or due to dietary and lifestyle constraints. For that purpose, we used a differential abundance analysis, applying a zero-inflated lognormal model in the package metagenomeSeq [43,44]. For relative abundances of bacterial genera, a generalized linear model with quasi-binomial distribution was used to model difference between the above-mentioned groups. Both models used the false discovery rate (fdr) to adjust *p*-values.

## 3. Results

### 3.1. Atopy- and breed-associated patterns in the gut microbiota

**Figure 1.**
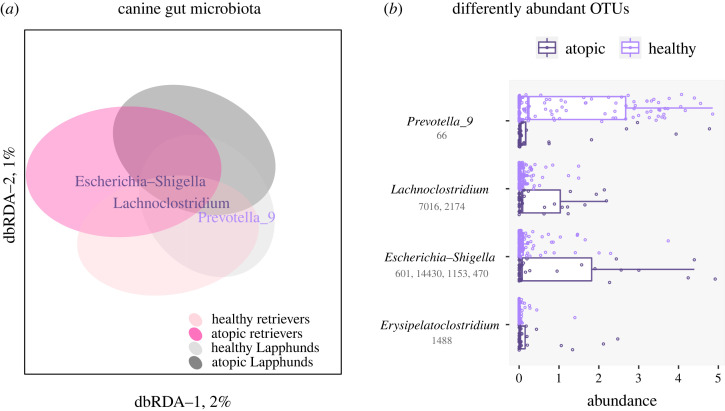
Relationships between breed, allergic symptoms and microbiota. (*a*) Compositional variation in microbiota between healthy and atopic Finnish Lapphund and Labrador Retrievers studied by distance-based redundancy analysis that used dogs' atopy status (atopic or healthy) and breed as constraining factors, and Bray–Curtis index to measure sample-wise differences. The ellipses show 0.75 confidence intervals of the group centroids. (*b*) Differently abundant (*p* ≤ 0.05) bacterial OTUs between healthy and atopic individuals. For visualization, CSS normalized OTU abundances were transformed by natural logarithm. *Prevotella_9* (Otu 66) was marginally significant (*p* = 0.11) but the pattern between atopic and healthy was distinct.

We investigated the role of dietary, lifestyle and environmental factors in the gut microbiota of pet dogs (table 1, electronic supplementary material, additional file 1, figure S1), considering potential association with atopy (i.e. owner-reported CAD). First, we questioned if dogs' gut microbiota composition and atopy were associated. In both breeds, Finnish Lapphunds and Labrador Retrievers, atopy was significantly associated with gut microbial composition that differed between healthy and atopic dogs (*p*_allergy_ = 0.019) (figure 1*a*). The association between microbiota and atopy could be found for OTUs, but no significantly different genera were observed. In healthy dogs (*n* = 125), an OTU of the genus *Prevotella_9* was more abundant, while in atopic dogs (*n* = 30), OTUs representing the genera *Escherichia-Shigella* were enriched (figure 1*b*). Otu66 of *Prevotella_9* was one of the major OTUs among all (*n* = 6382, of which 1473 OTUs were prevalent in at least 5% of samples and were included in the statistics) with a mean global relative abundance of 0.87% (0.94% in healthy, 0.61% in atopic). Only 17 OTUs were more abundant (a mean global relative abundance between 0.9 and 8.8%) than Otu66 of *Prevotella_9*. OTUs of *Escherichia-Shigella* were moderately abundant, together gathering a mean relative abundance of 0.24% (0.05% in healthy, 1.03% in atopic). Several OTUs annotated as *Escherichia-Shigella* were also significantly more abundant in those dogs that had been given antibiotics (*n* = 19, electronic supplementary material, additional file 1, figure S3). As 50% of atopic dogs, but only 3% of healthy dogs, had received antibiotics during their lifetime, the association between increasing abundance of *Escherichia-Shigella* and antibiotics could not be excluded.

**Table 1.** Characteristics of the dogs (*n* = 155) that participated in the study.

properties		participants (*n*)		
		both breeds	Finnish Lapphunds	Labrador Retrievers
individuals		155	96	56
sex	female	104	60	44
	male	51	36	15
family relations	mother	34	22	12
	offspring	121	74	47
atopy	atopic	30	15	15
	healthy	125	81	44
antibiotic use	never	136	90	46
	once or more	19	6	13
diet^a^	NHeP-LC-R	16	9	7
	HeP-HC-D	107	63	44
	HeP-HC-M	2	2	0
	mixed	21	17	4
	NA	9	5	4
lifestyle^b^	rural	66	52	14
	urban	89	44	45
living environment^b^	rural	86	52	34
	urban	69	44	25

NHeP-LC-R = non-heat-processed low-carbohydrate raw, HeP-HC-D = heat-processed high-carbohydrate dry, HeP-HC-M = heat-processed high-carbohydrate moist items in diet.

^a^A few dogs were fed exclusively NHeP-LC-R, HeP-HC-D or HeP-HC-M items. Thus, groups were formed based on being composed of one major component (greater than 33.3%) among NHeP-LC-R, HeP-HC-D or HeP-HC-M items, or no major component (mixed). Due to different group sizes, we used the percentage of NHeP-LC-R as a continuous variable in our statistical analyses, as it best explained the variation in the gut microbiota (electronic supplementary material, figure S2).

^b^Rural and urban lifestyle categories were formed by dividing the first principal coordinate (PCoA-1, electronic supplementary material, figure S1), summarizing different land-use types and lifestyle factors, respectively, to positive scores (greater than 0, rural) and negative scores (less than 0, urban).

Secondly, to understand the role of breed in the gut microbial compositions, we characterized the association between breed and the gut microbiota. The gut microbiota composition clearly differed between breeds (*p*_breed_ = 0.0012) (figure 1*a*). In line with that, breed was the main source of the inter-individual variation in microbiota (15.7%), which was significantly higher among atopic Labrador Retrievers than atopic Finnish Lapphunds (electronic supplementary material, additional file 1, figure S4a). The severity of atopic symptoms seemed to increase the inter-individual variation in the gut microbiota, explaining the high variation among Labrador Retrievers, which were generally more atopic and more medicated by antibiotics than Lapphunds (electronic supplementary material, additional file 1, figure S4).

In general, many of the studied dietary, lifestyle and environmental factors seemed to be associated with the dog breeds and their microbiota, but in different ways. For instance, diet was associated differently with the gut microbiota of Finnish Lapphunds and Labrador Retrievers (table 2). As the gut microbiota and associated factors distinguished between the breeds, we investigated atopy—associated and coinciding dietary, lifestyle and environmental patterns in the microbiota—separately for both breeds.

**Table 2.** Effects of the most significant explanatory variables on the canine gut microbiota.

	both breeds^a^ (*n* = 145)		Finnish Lapphund (*n* = 90)		Labrador Retrievers (*n* = 55)	
variable^b^	*R*^2^	*p*	*R*^2^	*p*	*R*^2^	*p*
dietary factors						
percentage of NHeP-LC-R^c^ items	0.021	**0.0002**	0.044	**0.0001**	0.025	0.11
lifestyle- and environmentally associated factors						
antibiotic use^d^	0.010	0.11	0.013	0.18	0.031	**0.032**
oral exposure to dirt	0.010	*0.067*	0.012	0.26	0.025	0.10

Significant *p* values and *p* values close to significance level (0.05) are marked in bold and italic, respectively.

**^a^**Datasets were combined but permutations were constrained within breeds.

**^b^**MRM model: microbiota∼percentage of NHeP-LC-R items in diet+antibiotics+oral exposure+lifestyle. Lifestyle was non-significant and excluded from the table.

**^c^**Non-heat-processed low carbohydrate raw.

**^d^**Antibiotics was a categorical variable describing whether dog has or has not been treated with antibiotic.

### 3.2. Diet played a central role in the gut microbiota of Finnish Lapphunds, while in Labrador Retrievers the microbiota was associated with atopy

**Figure 2.**
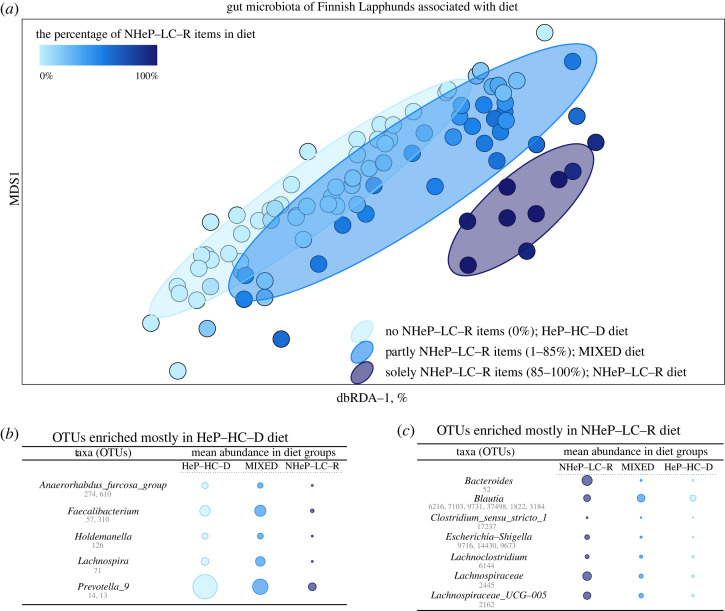
The gut microbiota of Finnish Lapphunds in relation to diet. (*a*) Changes in microbiota, which were due to differences in the content of non-heat-processed low-carbohydrate raw (NHeP-LC-R) items in dogs’ diet, were determined by distance-based redundancy analysis. Bray–Curtis index was used to measure sample-wise differences in the microbiota. The ellipses show 0.75 confidence intervals of the group centroids. The effects of other dietary factors such as probiotics, fats and heat-processed high-carbohydrate moist food on microbiota were minor but were taken into consideration. In practice, residual variation remaining from the fitting of these background factors were constrained by the percentage of NHeP-LC-R food. (*b*,*c*) Differently abundant (*p* < 0.05) OTUs that increased concurrently with the content of (*b*) heat-processed high carbohydrate dry (HeP-HC-D) items and (*c*) NHeP-LC-R items in diet. The colours of three groups are the same as in panel (*a*). Mean abundances of OTUs are based on the square-root-transformed CSS-normalized read counts.

Among all studied factors, the gut microbiota of Finnish Lapphunds was most significantly associated with the amount of NHeP-LC-R in diet (figure 2*a*, table 2, electronic supplementary material, additional file 1, figure S5). The NHeP-LC-R diet was associated with the increased abundance of OTUs belonging to the families Lachnospiraceae and Lachnospiraceae_UCG-005 and the genus *Bacteroides*. In turn, OTUs of some genera, such as *Faecalibacterium*, were almost completely missing in the gut of the Finnish Lapphunds that were on the NHeP-LC-R diet (figure 2*b*), but were highly abundant in those individuals on a HeP-HC-D diet. On the genus level, *Fusobacterium* increased and *Prevotella_9* as well as *Faecalibacterium* decreased (*p* < 0.01) along with the increasing proportion of NHeP-LC-R in diet (electronic supplementary material, additional file 1, figure S5). Diet and allergic symptoms were not statistically associated in Finnish Lapphunds.

**Figure 3.**
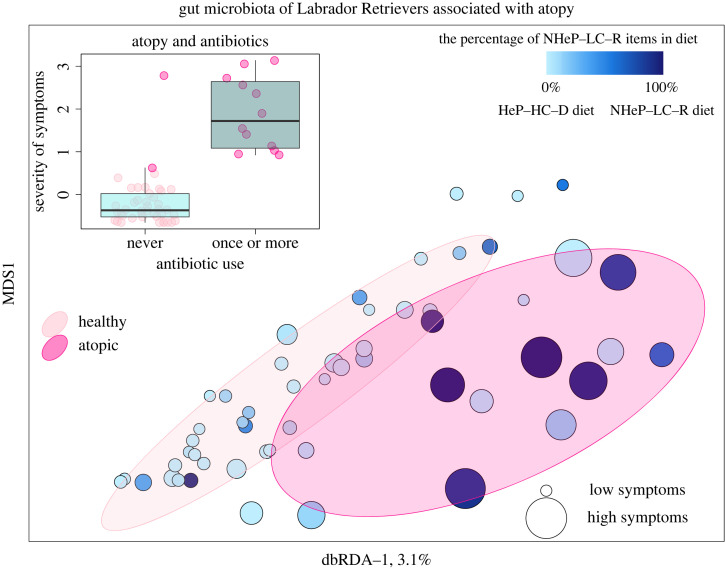
The gut microbiota of atopic and healthy Labrador Retrievers in relation to diet and antibiotic use. Compositional variation in microbiota was constrained by distance-based redundancy analysis that used severity of symptoms as an explanatory variable and Bray–Curtis index to measure sample-wise differences in the microbiota. An increasing size of a symbol indicates more severe atopy symptoms of the corresponding dog. Collinear factors, i.e. the percentage of non-heated low carbohydrate raw (NHeP-LC-R) items in dogs' diet and especially use of antibiotics increased along with the atopy symptoms.

In Labrador Retrievers, the severity of atopic symptoms (figure 3, *p* = 0.032) and the use of antibiotics (table 2, electronic supplementary material, additional file 1, figure S5) were associated with the gut microbiota clearly stronger than diet (table 2). The severity of atopic symptoms and antibiotic use were tightly coupled, whereas diet and symptoms were weakly associated. Labrador Retrievers with the most severe symptoms had received antibiotics (figure 3), and had been, in some cases, on the NHeP-LC-R diet (figure 3, electronic supplementary material, additional file 1, figure S1b). These associations prevent complete distinction of compositional changes in the microbiota attributed to diet, medication or allergy. However, *Escherichia-Shigella* was highly enriched in atopic dogs (figure 1*b*) or dogs treated with antibiotics (electronic supplementary material, additional file 1, figure S3) but only slightly enriched by diet (figure 2*c*).

Alpha diversity, measured as Shannon diversity index, was associated with the amount of NHeP-LC-R in the diet (*p* = 0.0003). The dogs having NHeP-LC-R items as the major components in their diet (greater than 33.3%) had lower diversity of amplicon sequencing variants (ASVs) in their gut microbiota than the dogs having less raw items in their diet (electronic supplementary material, additional file 1, figure S6). Alpha diversity was not significantly associated to any other studied factors.

### 3.3. The role of non-dietary lifestyle factors in canine atopy

Some dogs tend to eat or taste ‘dirt’ outside and thus are exposed to environmental or faecal microbes. Oral exposure to dirt, i.e. if dogs were allowed to eat or taste soil and faeces, drink muddy water etc., tended to be associated with gut microbial composition (table 2). Still, atopy was not significantly related to oral exposure to dirt in our data. The lifestyle variable, summarizing several other aspects of urban and rural lifestyle, and living environment (land-use) was not associated with the gut microbial composition.

## 4. Discussion

Using a canine model, we investigated the associations of lifestyle and environmental factors—including diet and antibiotics—on the gut microbiota of atopic and healthy pet dogs of two different breeds. Our results suggest that the gut microbial composition is contrastingly different between atopic and healthy dogs as well as dogs on different diets. Recent investigations suggest that changes in the human gut microbiota induced by a Western lifestyle, e.g. dietary shift to ‘high-fat, high-protein and high-sugar diet’ [20], are associated with allergies due to altered immune function, e.g. via less short-chain fatty acids (SCFAs) produced from diet by gut microbes [4,8]. Some allergy-associated gut microbial signatures have been identified in humans, either preceding the disease [49,50] or after the onset of the disease [51–53]. Information on how and which gut microbes are associated with allergy or atopy (AD) in dogs is scarce and from small samples [25,26].

In general, diet is a major determinant of the mammalian gut microbial composition [27,28,54]. As expected, we also detected a clear difference in the gut microbiota of dogs that were on different diets. Among all dietary factors considered, NHeP-LC-R diet was most significantly related to the composition of the gut microbiota, particularly in Finnish Lapphunds. The consumption of NHeP-LC-R food, containing raw items with more animal proteins and fat and less carbohydrates, associated with the enrichment of *Bacteroides* and Lachnospiraceae in the gut. The consumption of HeP-HC-D food, rich in carbohydrates [23], is in turn associated with the increased abundances of *Prevotella* and *Faecalibacterium*. These compositional changes are well in line with previous reports, illustrating that *Bacteroides* is related to the consumption of animal proteins and fats, while *Prevotella* and *Faecalibacterium* indicate carbohydrate-based diet and fibres, both in dogs [28,55,56] and humans [57,58].

Do these observed diet-associated differences in the microbiota contribute to or protect from atopy, or on broader perspective, allergies? In human societies, Western diet is rich in processed food, which has repeatedly been associated with elevated allergy risk [4,8]. Processed dog food has also become very popular, paralleling the increase in canine allergies [24,30]. Considering these coincidental phenomena, it is relevant to assume that dietary changes may be involved also in dogs' allergic diseases. However, contrary to our expectations, dogs that consumed HeP-HC-D food were mostly healthy. The genera *Prevotella* and *Faecalibacterium* that were enriched in their gut, are known to produce SCFAs from dietary fibres and carbohydrates [59,60].

SCFAs, although not measured in our study, modulate immune tolerance of the host [61–63]. Lower concentration of SCFAs have been detected in humans suffering from atopic dermatitis (AD), as compared with subjects without AD [60]. However, dogs do not rely on the microbial fermentation of carbohydrates as much as humans [23]. In addition, the knowledge on the role of SCFAs in canine immune function is scarce, and relationships between SCFAs, diets as well as microbiota rarely studied and contradictory [56,64]. Thus, whether the aforementioned microbes have allergy/atopy-protecting capacity, e.g. via SCFAs, remains an open question that should be studied in controlled conditions.

We found that the genus *Prevotella*_9 characterized the microbiota of healthy dogs while the genera *Escherichia-Shigella* was common in atopic dogs. In humans, the abundance of *Prevotella* has been negatively associated with some food allergies [52], while promoting several autoimmune diseases [65]. *Escherichia-Shigella*, in turn, has been associated with allergies in humans [50,53]. Thus, it seems, that *Escherichia-Shigella* indicates diseased conditions while *Prevotella*_9 may or may not be protective. *Escherichia-Shigella* increased also in the gut of dogs treated with antibiotics. As antibiotic use and atopic symptoms were associated, the exclusive designation of atopy-associated gut microbiota was not possible to report. As we received incomplete information on whether the antibiotics were given before or after the pet started to become atopic, we cannot either conclude that antibiotic treatment would play a predisposing role in CAD, even though this is likely, based on the existing literature. Antibiotics facilitate dysbiosis of the gut microbiota, which in turn can predispose to inflammatory disorders [5,66]. In terms of atopy, an overall risk increment of 41% for the development of AD has been reported for human subjects receiving even one course of antibiotics in early life [67]. Here, antibiotics were a source of inter-individual variation in microbiota, indicating perturbation to the gut microbiota.

As the gut microbiota is also controlled by genetic factors [68,69], one might expect that the genetic determinants of atopy could affect microbial communities. Here, the compositional differences and inter-individual variation in microbiota, as well as different atopy prevalence between breeds, indicate some genetic contribution. This is supported by studies showing different genetic risk for atopy in dog breeds [34,70,71]. Whether the gut microbiota of atopic individuals represent a proxy of their lifestyle, genetic factors, or atopic conditions *per se* would be important to distinguish, in order to treat atopy by manipulating the gut microbiota [72]. Nevertheless, the source for atopic-associated microbial signatures could not be traced here. Neither could it be assessed how socio-economic status of dog owners in terms of household income, education and other related aspects may have influence on the resources and interest in their pet's health, and thus generalizability of our results.

In microbiome research, animal models are useful for testing causalities and gaining more mechanistic understanding, as human microbial communities tend to represent high inter-individual variation (e.g. due to the large variation in human lifestyles). For studying relationships between lifestyle-associated microbiota and human atopy, pet dogs have several advantages. For example, canine AD shares several characteristics in common with human AD [46,73]. Importantly, dogs share the residential environment and lifestyle, sometimes even diet, with humans, contrary to laboratory rodent models. Lifestyle factors, predisposing dogs and humans to atopy, can be the same, and atopy in dogs and their owners can occur concurrently [16], which was also recently demonstrated [74] for the dogs of this study. An atopic dog more likely has an atopic than healthy owner. Lately, Coelho *et al*. [28] also showed that the dietary responses of the canine gut microbiota are comparable to those observed in humans. These previous observations are in line with ours. We also showed that the pet dogs' gut microbiota reflects the use of antibiotics, as found in the human gut microbiota, and diverged between atopic and healthy individuals. Our observations with the previous findings suggest that pet dogs could be a promising model for understanding associations of atopy with gut microbiota and related external factors.

## 5. Conclusion

Our study, utilizing privately owned pet dogs, demonstrates an interplay between dietary factors and antibiotic use, the prevalence of atopy and gut microbiota. The gut microbiota tends to be strongly affected by the type of diet. In addition, the microbiota diverged between atopic and healthy individuals. This is probably due to the differences in their lifestyle, such as the frequent usage of antibiotics in atopic dogs. *Escherichia-Shigella,* enriched by antibiotic use, seems to be a potential atopy-contributing candidate, which should be further tested in experimental set-ups. Importantly, our results are consistent with the observations from human studies showing that the gut microbiota reflects dietary and medical factors and the allergy prevalence of humans. Therefore, our results are partly applicable to humans and support the use of the canine model in exploring aetiology of atopic and allergic diseases. An interesting future direction would be to include genome-wide genetic variants to explore associations between diet and other lifestyle, genetic risk, allergy or atopy prevalence and microbiota.

## Data Availability

The 16S rRNA sequences supporting the results of this article are available in the NCBI SRA under the accession SRP152712. The normalized OTU counts and related taxonomy, metadata and the variables used in the statistical analyses are accessed as electronic supplementary material, additional file 2. R scripts for statistical analyses are included as additional files 3, 4 and 5. These files were created using the R package knitr [75]. The data are provided in the electronic supplementary material [76].
